# Supplementary material for: Baseline eGFR cutoff for increased risk of post-contrast acute kidney injury in patients undergoing percutaneous coronary intervention for ST-elevation myocardial infarction in the emergency department
Source: PLoS One. 2023 Oct 26;18(10):e0293598. doi: 10.1371/journal.pone.0293598 (PMC10602274; doi:10.1371/journal.pone.0293598)
Supplement: S1 File — (DOCX) [file pone.0293598.s001.docx]

**[Supplementary 1]**

**Table S1. Survival discharge according to PC-AKI occurrence**

|  | | Survival discharge | | | | Univariate | | Multivariate 1 | | Multivariate 2 | |
| --- | --- | --- | --- | --- | --- | --- | --- | --- | --- | --- | --- |
|  |  | No (n=34) | | Yes (n=720) | | Odds Ratio (95% CI) | | Odds Ratio (95% CI) | | Odds Ratio (95% CI) | |
|  | | N  or mean | SD or % | N  or mean | SD or % | OR | *p*-value | OR | *p*-value | OR | *p*-value |
| Previous  PC-AKI | No | 16 | 47.06 | 676 | 93.89 | 0.058 (0.028,0.121) | <.001 | 0.169 (0.066,0.433) | 0.001 |  |  |
|  | Yes | 18 | 52.94 | 44 | 6.11 | ref |  | ref |  |  |  |
| Current PC-AKI | No | 17 | 50.00 | 678 | 94.17 | 0.062 (0.030,0.130) | <.001 |  |  | 0.251 (0.091,0.691) | 0.007 |
|  | Yes | 17 | 50.00 | 42 | 5.83 | ref |  |  |  | ref |  |
| Baseline eGFR |  | 62.18 | 22.94 | 86.29 | 22.91 | 1.038 (1.024,1.053) | <.001 |  |  |  |  |
| eGFR group  (mL/min/1.73 m^2^) | <30 | 2 | 5.88 | 13 | 1.81 | 0.139  (0.027,0.720) | 0.1561 | 1.416  (0.218,9.190) | 0.772 | 1.641  (0.252,10.662) | 0.6536 |
|  | 30-59 | 16 | 47.06 | 85 | 11.81 | 0.114  (0.047,0.274) | 0.001 | 0.892  (0.279,2.844) | 0.476 | 0.957 (0.282,3.244) | 0.520 |
|  | 60-90 | 8 | 23.53 | 248 | 34.44 | 0.663  (0.246,1.790) | 0.036 | 1.493  (0.471,4.734) | 0.548 | 1.452  (0.463,4.555) | 0.683 |
|  | >=90 | 8 | 23.53 | 374 | 51.94 | ref |  | ref |  | ref |  |
| eGFR group 2  (mL/min/1.73 m^2^) | <60 | 18 | 52.94 | 98 | 13.61 | ref |  |  |  |  |  |
|  | >=60 | 16 | 47.06 | 622 | 86.39 | 0.140  (0.069,0.284) | <.001 |  |  |  |  |
| Age |  | 72.15 | 10.87 | 63.12 | 13.36 | 0.944  (0.917,0.973) | 0.001 |  |  |  |  |
| Age | <65 | 8 | 23.53 | 380 | 52.78 | ref |  |  |  |  |  |
|  | >=65 | 26 | 76.47 | 340 | 47.22 | 0.275  (0.123,0.616) | 0.002 |  |  |  |  |
| Age | <75 | 17 | 50.00 | 556 | 77.22 | ref |  |  |  |  |  |
|  | >=75 | 17 | 50.00 | 164 | 22.78 | 0.295  (0.147,0.591) | 0.001 |  |  |  |  |
| Sex | female | 15 | 44.12 | 158 | 21.94 | ref |  |  |  |  |  |
|  | male | 19 | 55.88 | 562 | 78.06 | 2.809  (1.396,5.654) | 0.004 |  |  |  |  |
| Hypertension | No | 14 | 41.18 | 324 | 45.00 | ref |  |  |  |  |  |
|  | Yes | 20 | 58.82 | 396 | 55.00 | 0.856  (0.425,1.720) | 0.662 |  |  |  |  |
| Diabetes mellitus | No | 22 | 64.71 | 532 | 73.89 | ref |  |  |  |  |  |
|  | Yes | 12 | 35.29 | 188 | 26.11 | 0.648  (0.314,1.335) | 0.239 |  |  |  |  |
| Hypercholesterolemia | No | 33 | 97.06 | 654 | 90.83 | ref |  |  |  |  |  |
|  | Yes | 1 | 2.94 | 66 | 9.17 | 3.330  (0.448,24.743) | 0.240 |  |  |  |  |
| Coronary artery Dz. | No | 32 | 94.12 | 620 | 86.11 | ref |  |  |  |  |  |
|  | Yes | 2 | 5.88 | 100 | 13.89 | 2.579  (0.609,10.928) | 0.198 |  |  |  |  |
| Heart failure | No | 34 | 100 | 711 | 98.75 | ref |  |  |  |  |  |
|  | Yes | 0 | 0 | 9 | 1.25 | 0.922  (0.045,18.881) | 0.958 |  |  |  |  |
| Arrhythmia | No | 32 | 94.12 | 702 | 97.50 | ref |  |  |  |  |  |
|  | Yes | 2 | 5.88 | 18 | 2.50 | 0.410  (0.091,1.845) | 0.245 |  |  |  |  |
| Stroke | No | 33 | 97.06 | 691 | 95.97 | ref |  |  |  |  |  |
|  | Yes | 1 | 2.94 | 29 | 4.03 | 1.384  (0.183,10.465) | 0.753 |  |  |  |  |
| Malignancy | No | 29 | 85.29 | 670 | 93.06 | ref |  |  |  |  |  |
|  | Yes | 5 | 14.71 | 50 | 6.94 | 0.433  (0.097,1.940) | 0.098 |  |  |  |  |
| Liver Dz | No | 34 | 100 | 715 | 99.31 | ref |  |  |  |  |  |
|  | Yes | 0 | 0 | 5 | 0.69 | 0.531  (0.022,12.897) | 0.697 |  |  |  |  |
| Chronic kidney Dz | No | 32 | 94.12 | 701 | 97.36 | ref |  |  |  |  |  |
|  | Yes | 2 | 5.88 | 19 | 2.64 | 0.433  (0.097,1.940) | 0.274 |  |  |  |  |
| LV EF (%) |  | 31.09 | 15.85 | 48.0 | 11.79 | 1.116  (1.082,1.151) | <.001 | 1.072  (1.034,1.110) | 0.001 | 1.070  (1.034,1.108) | 0.001 |
| Contrast amount (ml) |  | 203.68 | 76.39 | 192.85 | 86.10 | 0.999  (0.994,1.004) | 0.589 |  |  |  |  |
| Type of contrast  medium | Scanulx | 9 | 47.37 | 259 | 59.95 | ref |  |  |  |  |  |
|  | Visipaque | 10 | 52.63 | 167 | 38.66 | 0.584  (0.237,1.438) | 0.242 |  |  |  |  |
|  | Xenetix | 0 | 0 | 6 | 1.39 | 0.476  (0.020,11.385) | 0.647 |  |  |  |  |
| PCI results | CAOD  1VD | 11 | 32.35 | 269 | 37.47 | ref |  |  |  |  |  |
|  | CAOD  2VD | 13 | 38.24 | 194 | 27.02 | 0.610  (0.268,1.391) | 0.240 |  |  |  |  |
|  | CAOD  3VD | 8 | 23.53 | 194 | 27.02 | 0.992  (0.392,2.511) | 0.986 |  |  |  |  |
|  | Normal & minimal CAOD | 2 | 5.88 | 61 | 8.50 | 1.247  (0.270,5.772) | 0.778 |  |  |  |  |
| Cardiogenic shock | No | 13 | 39.39 | 601 | 84.29 | ref |  | ref |  | ref |  |
|  | Yes | 20 | 60.61 | 112 | 15.71 | 0.121  (0.059,0.251) | <.001 | 0.508  (0.205,1.262) | 0.145 | 0.418  (0.175,0.996) | 0.049 |
| Laboratory data |  |  |  |  |  |  |  |  |  |  |  |
| W.B.C. (10^3/μL) |  | 10.03 | 3.14 | 10.67 | 3.94 | 1.047  (0.950,1.154) | 0.351 |  |  |  |  |
| Hemoglobin (g/dL) |  | 13.93 | 1.81 | 14.16 | 2.07 | 1.054  (0.896,1.239) | 0.525 |  |  |  |  |
| Hematocrit (%) |  | 41.18 | 4.89 | 41.97 | 5.81 | 1.023  (0.966,1.084) | 0.432 |  |  |  |  |
| Delta neutrophil index (%) |  | 0.51 | 0.84 | 0.62 | 1.56 | 1.064  (0.793,1.429) | 0.678 |  |  |  |  |
| Platelet (10^3/μL) |  | 228.12 | 68.55 | 246.16 | 87.71 | 1.003  (0.998,1.008) | 0.229 |  |  |  |  |
| Neutrophil (%) |  | 63.12 | 17.59 | 65.65 | 16.62 | 1.009  (0.989,1.029) | 0.388 |  |  |  |  |
| Lymphocyte (%) |  | 27.69 | 15.16 | 25.64 | 14.89 | 0.991  (0.969,1.013) | 0.434 |  |  |  |  |
| Glucose (mg/dL) |  | 154.15 | 61.30 | 183.61 | 81.70 | 1.007  (1.000,1.013) | 0.039 |  |  |  |  |
| BUN (mg/dL) |  | 17.91 | 5.92 | 19.45 | 9.51 | 1.023  (0.976,1.072) | 0.348 |  |  |  |  |
| Uric acid (mg/dL) |  | 5.41 | 1.52 | 5.74 | 1.74 | 1.125  (0.911,1.389) | 0.275 |  |  |  |  |
| Total cholesterol (mg/dL) |  | 198.25 | 44.07 | 186.76 | 49.80 | 0.996  (0.986,1.005) | 0.363 |  |  |  |  |
| Creatinine kinase (U/L) |  | 256 | 316 | 370 | 691 | 1.040  (0.958,1.129) | 0.348 |  |  |  |  |
| CK-MB (ng/mL) |  | 18 | 34 | 27 | 66 | 1.407  (0.638,3.104) | 0.398 |  |  |  |  |
| Troponin T (pg/mL) |  | 198 | 355 | 459 | 1319 | 1.039  (0.972,1.111) | 0.260 |  |  |  |  |
| NT pro BNP (pg/mL) |  | 1754 | 3155 | 2051 | 5303 | 1.001  (0.994,1.009) | 0.751 |  |  |  |  |
| Triglyceride (mg/dL) |  | 131.72 | 99.86 | 122.32 | 98.35 | 0.999  (0.996,1.002) | 0.615 |  |  |  |  |
| HDL-Cholesterol (mg/dL) |  | 42.87 | 10.73 | 40.69 | 10.56 | 0.982  (0.950,1.0140 | 0.269 |  |  |  |  |
| LDL-Cholesterol (mg/dL) |  | 91.33 | 39.90 | 100.48 | 47.23 | 1.006  (0.996,1.016) | 0.255 |  |  |  |  |
| CRP (mg/L) |  | 14.73 | 24.90 | 22.30 | 45.11 | 1.006  (0.994,1.018) | 0.348 |  |  |  |  |
| Use of nephrotoxic medication |  |  |  |  |  |  |  |  |  |  |  |
| Before ACEI/ARB | No | 29 | 90.63 | 605 | 86.80 | ref |  |  |  |  |  |
|  | Yes | 3 | 9.38 | 92 | 13.20 | 1.470  (0.439,4.923) | 0.532 |  |  |  |  |
| Before Beta blocker | No | 29 | 90.63 | 650 | 93.26 | ref |  |  |  |  |  |
|  | YES | 3 | 9.38 | 47 | 6.74 | 0.699  (0.205,2.379) | 0.567 |  |  |  |  |
| Before Statin | No | 28 | 87.50 | 558 | 80.06 | ref |  |  |  |  |  |
|  | Yes | 4 | 12.50 | 139 | 19.94 | 1.744  (0.602,5.053) | 0.306 |  |  |  |  |
| Before Insulin | No | 32 | 100 | 691 | 99.14 | ref |  |  |  |  |  |
|  | Yes | 0 | 0 | 6 | 0.86 | 0.611  (0.027,13.944) | 0.758 |  |  |  |  |
| Before oral DM | No | 25 | 78.13 | 574 | 82.35 | ref |  |  |  |  |  |
|  | Yes | 7 | 21.88 | 123 | 17.65 | 0.765  (0.324,1.809) | 0.542 |  |  |  |  |
| Before NSAID | No | 32 | 100 | 688 | 98.85 | ref |  |  |  |  |  |
|  | Yes | 0 | 0 | 8 | 1.15 | 0.802  (0.038,16.844) | 0.887 |  |  |  |  |
| After ACEI /ARB | No | 31 | 91.18 | 256 | 35.56 | ref |  | ref |  | ref |  |
|  | Yes | 3 | 8.82 | 464 | 64.44 | 18.729  (5.670,61.864) | <.001 | 8.247  (2.264,30.044) | 0.001 | 8.053  (2.235,29.017) | 0.001 |
| After Beta blocker | No | 32 | 94.12 | 582 | 80.83 | ref |  |  |  |  |  |
|  | Yes | 2 | 5.88 | 138 | 19.17 | 3.794  (0.898,16.021) | 0.070 |  |  |  |  |
| After Statin | No | 4 | 11.76 | 33 | 4.58 | ref |  |  |  |  |  |
|  | Yes | 30 | 88.24 | 687 | 95.42 | 2.776  (0.924,8.340) | 0.069 |  |  |  |  |
| After Insulin | No | 23 | 67.65 | 636 | 88.33 | ref |  |  |  |  |  |
|  | Yes | 11 | 32.35 | 84 | 11.67 | 0.276  (0.130,0.587) | 0.001 |  |  |  |  |
| After oral DM | No | 32 | 94.12 | 627 | 87.08 | ref |  |  |  |  |  |
|  | Yes | 2 | 5.88 | 93 | 12.92 | 2.373  (0.559,10.063) | 0.241 |  |  |  |  |
| After NSAID | No | 34 | 100 | 709 | 98.47 | ref |  |  |  |  |  |
|  | Yes | 0 | 0 | 11 | 1.53 | 1.118  (0.057,21.933) | 0.942 |  |  |  |  |

**Abbreviations :** PC-AKI, post contrast- acute kidney injury; CI, confidence interval; SD, standard deviation; OR. Odds ratio; eGFR, estimated glomerular filtration rate; Dz, disease; LV EF, left ventricular ejection fraction; PCI, percutaneous coronary intervention; CAOD, coronary artery obstructive disease; VD, vessel disease; W.B.C., White blood cell; BUN: blood urea nitrogen; CK, Creatinine kinase; NT pro-BNP, N-terminal pro-B-type natriuretic peptide; HDL, high density lipoprotein; LDL, low density lipoprotein; CRP,C-reactive protein; ACEI, angiotensin-converting enzyme inhibitor; ARB, angiotensin receptor blocker; DM, Diabetes mellitus; NSAID, nonsteroidal anti-inflammatory drug.
