# Supplementary material for: Baseline eGFR cutoff for increased risk of post-contrast acute kidney injury in patients undergoing percutaneous coronary intervention for ST-elevation myocardial infarction in the emergency department
Source: PLoS One. 2023 Oct 26;18(10):e0293598. doi: 10.1371/journal.pone.0293598 (PMC10602274; doi:10.1371/journal.pone.0293598)
Supplement: Attachment — Submitted filename: Review of the article.docx [file pone.0293598.s003.docx]

Review of the article

In the present paper, the authors present their data of optimal cut-offs of GFR for predicting CI-AKI.

The introduction can be better rephrased. Following are some point regarding the same.

**Comment 1**: **line 51:** **pPCI is a high-risk procedure for post-contrast acute kidney injury (PC-AKI).** Here we can modify as - ACS is a high risk state due to highly thrombogenic state, increased inflammation, and decreased renal perfusion through vasoconstriction or hemodynamic instability. Where exposure of contrast medium can increase chances of CI AKI. Authors can give quotations of studies reporting such increased incidences of AKI in pPCI.

**Comment 2**: line 56: Unlike CT- kindly use full form

**Comment 3**: for PCI, the **medium: use contrast**

**Comment 4: ‘**Although some reports suggest that hydration can reduce PC-AKI in patients with STEMI, to our knowledge, there are no effective therapeutic modalities for the prevention of PC-AKI.’ – Pre and post PCI hydration is the only intervention which has shown to reduce incidences of CI-AKI. Kindly give citiations too.

**Comment 5**: ‘Studies have shown that certain patient groups with baseline renal function, such as those with estimated glomerular filtration rates (eGFRs) <30, <45, or <60, are at an increased risk of developing PC-AKI’ – Kindly use 1 cutoff and their citation. Mostly <60 has been universally accepted as upper cut-off.

**Comment 6**: line 304: Hypotension : cardiogenic shock

**Comment 7**: Line 324 ‘The fact that there was no significant difference in the contrast mediums amount between the group with PC-AKI and the group without PC-AKI in this study also supports these results’ – This conclusion is oversimplification of results. Studies have proved that the lower the contrast we use the less are the chances of developing CI-AKI and thus the advent of ultra-low contrast PCI in such high risk low GFR cases. There have been recently more robust data published which further strengthen the case for a ULC-PCI. PMID: 36007555. These data can be added to further enhance the evidence base for the same and improve the impact of the article.

**Comment 8**: Addition of pre-procedural risk scores and post-procedure risk scores can be done which help objectively in decision making regarding the risk of the patient before PCI. The patietts could have been classified according to their risk scores and would have given a more deeper insight on the causes of development od AKI in an individual.

**Comment 9:** The average contrast consumption in the setting of pPCI was more than 100 ml which is acceptable in a pPCI setting. However what do the authors conclude in a stting of a low GFR what should the interventionalist do. Should he give less contrast or give more hydration? This articles conclusion seems there is no association with the amount of contrast use so such an exercise would prove futile. Kindly elaborate in discussion as to what this article and these cut-offs mean in real world.

**Comment 10**: Also what was the follow-up GFR of patients post intervention. Does primary PCI help the GFR in the longer run. A longer follow-up would have helped. As reperfusion helps improve cardiac hemodynamics and renal hemodynamics in the patients. So is pPCI benificial to the patient renal wise . what are authors opinions regarding it.

**Comment 11**: what are authors views after the study as to which patients should receive ACEi and ARNis and on what day after intervention based on their data. Any expert comments as to minimize the risk of AKI and also utilize them for their benifits
