# Supplementary material for: Baseline eGFR cutoff for increased risk of post-contrast acute kidney injury in patients undergoing percutaneous coronary intervention for ST-elevation myocardial infarction in the emergency department
Source: PLoS One. 2023 Oct 26;18(10):e0293598. doi: 10.1371/journal.pone.0293598 (PMC10602274; doi:10.1371/journal.pone.0293598)
Supplement: Attachment — Submitted filename: Response_to_reviewers (3).docx [file pone.0293598.s005.docx]

**Additional Editor Comments:**

***Response***: We appreciate your insightful comments and useful suggestions regarding our manuscript. Based on these suggestions, we have thoroughly revised our manuscript. Please find point-by-point responses to all individual comments below.

Dear authors,

Thank you for your kind response and effort on the review process. Your manuscript has improved significantly. However, I have only one concern. The original data was acquired prospectively; therefore, written informed consent should be obtained. Please clarify whether the investigators have obtain consent from patients.

***Response:*** We apologize for any confusion caused. As stated in the Materials and Methos sections of the manuscript, this is a retrospective observational cohort study. We retrospectively analyzed data from a registry; thus, the need for informed consent was waived. However, we employed the concept of a prospective registry to explain that the activation of critical pathways was prospectively and sequentially performed based on a predetermined protocol when emergent patients with STEMI visited the emergency department, and that these data were accumulated in electronic health records (EMR). When this critical pathway was initially implemented in the emergency department, patient consent was not required because the data were collected in advance solely for the critical clinical care of the patient, rather than for research purposes. We appreciate your feedback and have removed the term “prospective” from the Materials and Methods section to avoid any potential confusion for the readers:

“This retrospective, observational cohort study used a ~~prospective~~ registry of a critical pathway in a single tertiary academic hospital that attends to 100,000 patients in the ED annually.”

**Reviewer #1**: The authors have completed a thoroughly review of their paper addressing almost all reviewers’ comments.
The paper has greatly improved now but there are still few minor issues to be addressed.

***Response***: We appreciate your kind comments and useful suggestions regarding our manuscript.

1. Door-to-balloons time are now presented as median and IQR and the authors state that there are no significant difference with a p-value of 0.45. Yet in table 1 the reported p-value is <0.001. Please re-check the variable and the gaussianity test to confirm the non-normal distribution and present it accordingly.

***Response:*** We apologize for this mistake. As you noted, we report the door-to-balloon time as median and interquartile range (IQR), and the p-value is <0.001, as shown in Table 1. We clarified the presentation of the data and the reference to Table 1 in the revised manuscript. The numbers described in the Table 1 in manuscript are correct, and the reviewer's response was submitted without modification, causing confusion. In conclusion, there is a higher probability of PC-AKI occurrence associated with longer door-to-balloon times, which was consistent with the results of our study.

2. In table 1, in the variable description along with the measurement units should be reported inside brackets whether a percentage or IQR or SD are reported. The same goes for header of table 2A.

***Response***: We appreciate this suggestion and have revised the Tables accordingly.

3. Line 131 door-to-balloon times are still reported as mean and SD. Please provide a uniform reporting according to comment #1.

***Response:*** We apologize for this mistake. The values have been revised to median (IQR) accordingly.

4. Line 313: a very simplified definition of cardiogenic shock is reported. I think the authors want to stress out the importance of hypoperfusion as a prominent risk factor for AKI. I suggest to remove the definition or use a more accurate one (SBP<90 mmHg for ≥30 min or use of pharmacological and/or mechanical support to maintain an SBP≥90 mmHg paired with evidence of end‐organ hypoperfusion which typically includes urine output<30 mL/h, cool extremities, altered mental status, and/or serum lactate >2.0 mmol/L.

***Response***: We appreciate this important suggestion. We have reviewed our data and revised the definition of cardiogenic shock in our manuscript according to your comment:

“Cardiogenic shock is defined as ~~a systolic blood pressure of < 80 mm Hg for more than 60 min~~ a systolic blood pressure (SBP) <90 mmHg for ≥30 min or the use of pharmacological and/or mechanical support to maintain an SBP ≥90 mmHg paired with evidence of end‐organ hypoperfusion, which is also a risk factor for PC-AKI.”

5. Line 322: the word “impaired” is missing from the sentence “ACEi in patients with … glomerular filtration”

***Response***: Thank you for pointing this out. We have added “impaired” to the revised manuscript:

“However, AKI can be induced by ACEIs in patients with impaired glomerular filtration, depending on the angiotensin II-mediated efferent vascular tone, for instance in patients with renal artery disease, heart failure, or severe volume depletion [27].”

6. Line 324: replace “renal arterial disease” with “renal artery disease”

***Response***: Thank you for pointing this out; we have revised the manuscript accordingly.

7. Line 370: the sentence has been truncated. Please add “factors for PC-AKI” after “several high-risk”

***Response***: Thank you for pointing this out. Accordingly, we have added “factors for PC-AKI” after “several high-risk” in the revised manuscript:

“The clinical implications of our results are significant, as they could inform clinical decisions on the use of contrast-enhanced imaging procedures for patients with reduced kidney function or several high-risk factors for PC-AKI.”

8. The final result of the multivariable analysis showed that eGFR and impaired cardiac output are the most important risk factors for AKI development in STEMI patients, regardless of previous medical history, medications and laboratory values. This finding is extremely important and should be emphasized. Following this concept a more detailed description of the hemodinamic status should be provided. It would be very interesting to compare between AKI and non-AKI patients the values of SBP, MBP, DBP, HR and if available also lactate levels. Those values can be measured at baseline, during or after the procedure and their significance can inform clinicians and interventional cardiologist about the proper management of the patient.

***Response***: We highly appreciate these comments and suggestions. According to these, we have performed further statistical analyses to compare systolic blood pressure (SBP), diastolic (DBP), and heart rate (HR) between AKI and non-AKI patients. Unfortunately, data on lactate levels were not separately collected in our study.

|  | No PC-AKI | PC-AKI | p-value |
| --- | --- | --- | --- |
| SBP (mmHg), mean (IQR) | 132 (112,152) | 130 (103,164) | 0.791 |
| DBP (mmHg), mean (IQR) | 80.5 (68,93) | 78 (65,91) | 0.479 |
| HR (pulse rate), mean (IQR) | 80 (68,94) | 93 (77,116) | 0.002 |

We found a significant difference in HR between AKI and non-AKI patients. However, there was no significant difference in SBP or DBP between the two groups. We hypothesize that there was no significant difference in SBP and DBP between the two groups, because these vital signs were recorded upon arrival at the emergency department. Unfortunately, due to the absence of predetermined time points for vital sign measurements in this study, we were unable to analyze hemodynamic changes after admission to the emergency department. To avoid this limitation caused by the retrospective nature of our study, conducting a prospective study to monitor hemodynamic changes, including blood pressure, heart rate, and lactate levels at predetermined time points, and comparing them between AKI and non-AKI patients would be a valuable avenue for future research.

9. Please provide the reasons for IABP implantation (high risk anatomy, impending shock and so on).

We have thoroughly discussed this matter with cardiologists at our hospital. Currently, our hospital does not have predetermined guidelines in place. Nevertheless, we generally implement IABP as follows:

In patients with STEMI accompanied by cardiogenic shock with high inotropic requirements, if ECMO is deemed excessive due to the patient’s alert consciousness, we proceed with PCI after inserting an IABP.

Despite those minor comments I think the work of Dr. You and colleagues is extremely valuable and they should be praised for providing such important information for a clinical condition still profoundly neglected.

**Reviewer #2**: I think the authors have adequately addressed ALL QUERIES FROM MY SIDE. the authors have delved in hard to provide in-depth insight into the subject

***Response***: We appreciate your kind comments and useful suggestions regarding our manuscript.
